# Directed cell migration in the presence of obstacles

**DOI:** 10.1186/1742-4682-4-2

**Published:** 2007-01-16

**Authors:** Ramon Grima

**Affiliations:** 1 Indiana University School of Informatics and Biocomplexity Institute, Bloomington, IN 47406, USA; 2 Institute for Mathematical Sciences, Imperial College, London SW7 2PG, UK

## Abstract

**Background:**

Chemotactic movement is a common feature of many cells and microscopic organisms. *In vivo*, chemotactic cells have to follow a chemotactic gradient and simultaneously avoid the numerous obstacles present in their migratory path towards the chemotactic source. It is not clear how cells detect and avoid obstacles, in particular whether they need a specialized biological mechanism to do so.

**Results:**

We propose that cells can sense the presence of obstacles and avoid them because obstacles interfere with the chemical field. We build a model to test this hypothesis and find that this naturally enables efficient at-a-distance sensing to be achieved with no need for a specific and active obstacle-sensing mechanism. We find that (i) the efficiency of obstacle avoidance depends strongly on whether the chemotactic chemical reacts or remains unabsorbed at the obstacle surface. In particular, it is found that chemotactic cells generally avoid absorbing barriers much more easily than non-absorbing ones. (ii) The typically low noise in a cell's motion hinders the ability to avoid obstacles. We also derive an expression estimating the typical distance traveled by chemotactic cells in a 3D random distribution of obstacles before capture; this is a measure of the distance over which chemotaxis is viable as a means of directing cells from one point to another *in vivo*.

**Conclusion:**

Chemotactic cells, in many cases, can avoid obstacles by simply following the spatially perturbed chemical gradients around obstacles. It is thus unlikely that they have developed specialized mechanisms to cope with environments having low to moderate concentrations of obstacles.

## Background

Directed cell motion is a common feature of many cells and micro-organisms; this movement can be induced by a number of factors including light (phototaxis), gravity (gravitotaxis) and various chemicals (chemotaxis). The last of these is the most pervasive natural form of taxis. The bacteria *Escherichia coli *and *Salmonella typhimurium*, the slime mould *Dictyostelium discoideum*, and neutrophils [1] are a few of the many well studied examples of chemotactic life-forms. Chemotaxis involves the detection of a local chemical gradient and the subsequent movement of the organism up (positive chemotaxis) or down (negative chemotaxis) the gradient. For example, *Dictyostelium discoideum *follows trails of folic acid secreted by its food source, bacteria, so as to track and eventually capture them [2]. Another example is the chemotaxis of neutrophils to gradients of C5a released at a wound site – the neutrophils kill bacteria and decontaminate the wound from foreign debris.

Over the years, various aspects of chemotactic behavior have been studied from both an experimental and a theoretical point of view (e.g. [3-9]). In this article we study the efficiency of chemotaxis in achieving controlled cell migration to specific sites in the heterogeneous environments typical of *in vivo *conditions. Current models of chemotactic movement do not directly address such issues. Typically, these models simulate the interaction of cells with each other and ignore the physical environment in which the movement is occurring, e.g. non-chemotactic cells and foreign debris in the path of the migrating chemotactic cells.

Since environmental heterogeneity occurs on a scale comparable to that of individual cells, macroscopic continuum models (usually based on the Keller-Segel model [10]) of cell movement are not appropriate to answer the above questions. Rather, one requires an approach involving an individual-based model (IBM) of cell movement. In this article we construct a minimal IBM of chemotactic cell movement in an obstacle-ridden environment. Our aim is to understand the efficiency of chemotaxis in such conditions and whether additional biological mechanisms (e.g. an active obstacle-sensing mechanism) are needed to ensure that the chemotactic cell reaches the source of the chemical to which it is sensitive. A few specialized mechanisms of this type are known, for example the case of axon guidance [11], in which a combination of chemoattractants and chemorepellents secreted by other cells in the environment guide the axons along very specific routes to generate precise patterns of neuronal wiring. However, this is not the general case, particularly for free-swimming cellular organisms, which may be simply involved in following chemoattractant left by their prey and thus have no apparent foreknowledge of any obstacles in their path. These are the cases we shall treat in this study.

In the next section, we first review and summarize some basic facts about cell movement that follow from the underlying biology and physics. On the basis of this information, we build the simplest (deterministic) realistic model of cell movement and by means of an analytical analysis we use it to understand the movement of a chemotactic cell in the presence of a single obstacle. This will clearly prove that cells can naturally sense and avoid obstacles by simply following the chemical gradient and that in many cases they do not require additional specialized mechanisms. We study the efficiency of obstacle avoidance as a function of the cell-obstacle size ratio and the type of obstacle: obstacles can either not interact with the chemotactic chemical or act as a sink. Next, we study the effect of noise on the probability of the cell being captured by an obstacle, and finally we conclude by extending our analysis to the case of a multi-obstacle environment. This leads to an expression for the distance over which chemotaxis is viable as a means of directing cells from one point to another *in vivo*.

### Chemotactic motion of a cell around an obstacle

In this section we study the motion of a chemotactic cell when a single obstacle is placed in its migratory path towards the chemoattractant source. There are two types of chemotactic sensing: (i) Spatial sensing, in which a cell compares the chemoattractant concentration at two different points on its body. This mechanism is, for example, used by the slime mould and neutrophils. (ii) Temporal sensing, in which a cell compares the concentration at two different times. This is used by flagellated bacteria such as *Escherichia coli *and *Salmonella typhimurium*. There is a process related to chemotaxis, called chemokinesis, in which the speed of cell movement is determined by the absolute value of the local concentration but the cell does not actually orient [12]. In this article we shall be concerned exclusively with chemotaxis via a spatial sensing mechanism.

### The non-absorbing obstacle case

**Figure 1:**
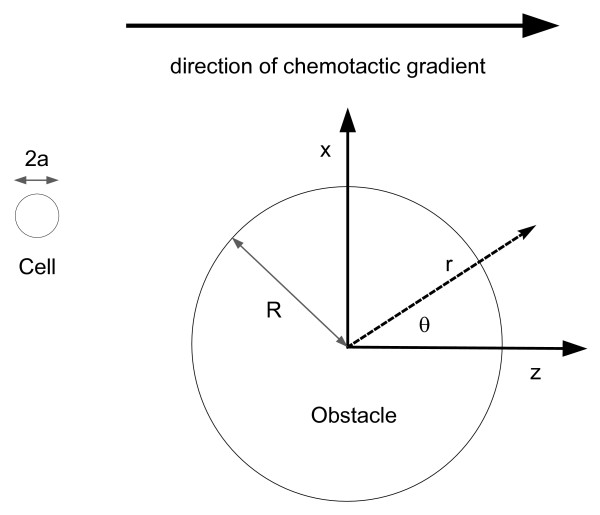
**Graphical representation of the system under investigation**. A spherical obstacle of radius R is placed in the path of a chemotactic cell of radius a. The chemical gradient far away from the obstacle is constant and in the z-axis direction. The y-axis is out of the page. The angle *θ *is measured anticlockwise from the positive z-axis.

Consider a spherical chemotactic cell of radius *a *in a uniform chemical gradient of magnitude ∇ *C *= *g *z^
 MathType@MTEF@5@5@+=feaafiart1ev1aaatCvAUfKttLearuWrP9MDH5MBPbIqV92AaeXatLxBI9gBaebbnrfifHhDYfgasaacH8akY=wiFfYdH8Gipec8Eeeu0xXdbba9frFj0=OqFfea0dXdd9vqai=hGuQ8kuc9pgc9s8qqaq=dirpe0xb9q8qiLsFr0=vr0=vr0dc8meaabaqaciaacaGaaeqabaqabeGadaaakeaacuWG6bGEgaqcaaaa@2E39@; the gradient is chosen to be directed along the positive z-axis in a right-handed coordinate system. Note that *C *denotes the chemical field. If the cell is positively chemotactic, its movement is up the gradient in a direction parallel to the z-axis. Next we introduce a spherical obstacle of radius *R *centered at the origin. The setup is illustrated in Fig. 1. The question we are interested in is: Can the cell avoid the obstacle just by following the chemical gradient or does it need an additional biological mechanism ? Note that the cell and the obstacle are assumed to be in a fluid at rest; the obstacle is stationary relative to the fluid and immobile; only the cell moves. We wish to make absolutely clear that this is not the classical case of a cell carried by a moving fluid past a stationary obstacle. The cell's movement is only due to its response to external chemotactic stimuli.

Now we proceed to construct a simple physical model to answer the above question. First we summarize some basic facts about cell movement, which follow from the underlying physics and/or biology:

1. Inertial effects are insignificant to the cell's movement. This follows from the fact that cells and micro-organisms typically exist in low-Reynold's number environments [13,14].

2. The cell is able to resolve the chemical gradient along its body (a spatial sensing mechanism). It is well known that many eukaryotic cells [12] and even some types of bacteria [15] have this ability.

3. The chemotactic force on a cell is directly proportional to the chemical gradient across its body. This is implicity assumed in many models of chemotaxis, such as the Keller-Segel model [10] and its discrete counterpart [8]. This approximation is satisfactory if the chemical concentration is not too large; this follows theoretically from a consideration of receptor kinetics (see [16] for example).

It thus follows that the cell's movement can be modeled via an over-damped equation of the form:

x˙
 MathType@MTEF@5@5@+=feaafiart1ev1aaatCvAUfKttLearuWrP9MDH5MBPbIqV92AaeXatLxBI9gBaebbnrfifHhDYfgasaacH8akY=wiFfYdH8Gipec8Eeeu0xXdbba9frFj0=OqFfea0dXdd9vqai=hGuQ8kuc9pgc9s8qqaq=dirpe0xb9q8qiLsFr0=vr0=vr0dc8meaabaqaciaacaGaaeqabaqabeGadaaakeaaieqacuWF4baEgaGaaaaa@2E34@_*c *_(*t*) = *α *∇ *C *(**x**_*c *_(*t*), *t*),     (1)

where **x**_*c *_(*t*) is the position of the cell's center of mass at time *t *and *α *is a positive constant measuring the cell's chemotactic sensitivity. Note that the above equation follows from Newton's second law when viscous drag dominates over the inertial force (i.e. small Reynold's number). For the moment we ignore stochastic contributions to the cell's trajectory; effects stemming from intrinsic noise will be studied in a later section. Note that in our mathematical formulation, the cell's motion is determined by the chemical gradient in the center of the cell. This is a good approximation to the gradient across their bodies (which is what is actually measured) provided the cell is not too large. Using the gradient at the center of the cell will enable a mathematical analysis to be conducted that is not possible otherwise; however, in our ensuing numerical simulations, we will compare results using both the gradient at the cell's center and that calculated as a concentration difference across the cell's body.

Next we need to specify equations for the chemical field. Two main considerations determine these equations:

1. The interaction of the chemical with the obstacle's surface. The object can be impermeable to the chemical, i.e. the chemical bounces off the obstacle's surface without any appreciable absorption, or it can interact with the chemical.

2. The diffusive relaxation time of the chemical field will determine whether the field sensed by the cell is in steady-state. For the sake of mathematical simplification, we shall assume that it is. This is physically justified in two cases: (i) the chemical field is set up well before cell migration starts. This is thought to be the case, for example, in some morphogenetic processes [17], where cells follow a chemical pre-pattern laid at an earlier time, (ii) If both the set up of the field and cell migration occur at the same time, then steady-state can only be achieved if the time taken to set up the field over the obstacle region by diffusion, Δ*t*_*D*_~*R*^2^/*D*_*c*_, is much less than the time taken for the cell to traverse the same region, Δ*t*_*c*_~*R*/*αg*. Note that *D*_*c *_is the chemical diffusion coefficient and that the last two expressions are correct up to some multiplicative constant. Thus the steady state assumption is valid if the inequality *αgR*/*D*_*c *_≪ 1 is approximately satisfied.

Given these considerations and assuming isotropy of the medium in which cell movement occurs (i.e. the chemical diffusion coefficient is not a function of space but a constant), the chemical field is described by Laplace's equation ∇^2 ^*C *(*r*, *θ*, *φ*) = 0, with boundary condition: ∇ C = *g *z^
 MathType@MTEF@5@5@+=feaafiart1ev1aaatCvAUfKttLearuWrP9MDH5MBPbIqV92AaeXatLxBI9gBaebbnrfifHhDYfgasaacH8akY=wiFfYdH8Gipec8Eeeu0xXdbba9frFj0=OqFfea0dXdd9vqai=hGuQ8kuc9pgc9s8qqaq=dirpe0xb9q8qiLsFr0=vr0=vr0dc8meaabaqaciaacaGaaeqabaqabeGadaaakeaacuWG6bGEgaqcaaaa@2E39@ in the limit *r *→ ∞. We note that there may be many situations *in vivo *when the isotropy assumption does not hold; we shall ignore such complications, though many of the results we shall derive probably also translate to cases where the properties of the medium change very slowly over the region in which the obstacle is located. In this subsection we shall treat the case of a non-absorbing obstacle, which follows by imposing the surface no-flux boundary condition ∇_*r *_*C *(*r *= *R*) = 0. In the next subsection we shall consider the opposite case of an absorbing obstacle.

Now that we have specified equations for both cell movement and the chemical field, we proceed to determine the cell's spatial trajectory. The obstacle's physical presence significantly distorts and modifies the chemical field in its surroundings and thus alters the cell's chemotactic movement. Adopting spherical coordinates, we solve Laplace's equation with the specified boundary conditions. Since the chemical gradient for large *r *is along the z-axis, the solution has to possess azimuthal symmetry (i.e. symmetric with respect to rotations in *φ*); then the general solution to Laplace's equation is:

C(r,θ,φ)=∑i=0∞(Alrl+Blr−(l+1))pl(cos⁡θ),
 MathType@MTEF@5@5@+=feaafiart1ev1aaatCvAUfKttLearuWrP9MDH5MBPbIqV92AaeXatLxBI9gBaebbnrfifHhDYfgasaacH8akY=wiFfYdH8Gipec8Eeeu0xXdbba9frFj0=OqFfea0dXdd9vqai=hGuQ8kuc9pgc9s8qqaq=dirpe0xb9q8qiLsFr0=vr0=vr0dc8meaabaqaciaacaGaaeqabaqabeGadaaakeaacqWGdbWqdaqadaqaaiabdkhaYjabcYcaSGGaciab=H7aXjabcYcaSiab=z8aMbGaayjkaiaawMcaaiabg2da9maaqahabaWaaeWaaeaacqWGbbqqdaWgaaWcbaGaemiBaWgabeaakiabdkhaYnaaCaaaleqabaGaemiBaWgaaOGaey4kaSIaemOqai0aaSbaaSqaaiabdYgaSbqabaGccqWGYbGCdaahaaWcbeqaaiabgkHiTmaabmaabaGaemiBaWMaey4kaSIaeGymaedacaGLOaGaayzkaaaaaaGccaGLOaGaayzkaaGaemiCaa3aaSbaaSqaaiabdYgaSbqabaGcdaqadaqaaiGbcogaJjabc+gaVjabcohaZjab=H7aXbGaayjkaiaawMcaaiabcYcaSaWcbaGaemyAaKMaeyypa0JaeGimaadabaGaeyOhIukaniabggHiLdaaaa@5B2B@

where *P*_*l *_are Legendre polynomials, *A*_*l *_and *B*_*l *_are constants to be determined from the boundary conditions and *l *is an integer. Imposing the boundary conditions, the chemical field in the space around the spherical obstacle is given by:

C(r,θ)=C0+grcos⁡θ(1+R32r3),     (2)
 MathType@MTEF@5@5@+=feaafiart1ev1aaatCvAUfKttLearuWrP9MDH5MBPbIqV92AaeXatLxBI9gBaebbnrfifHhDYfgasaacH8akY=wiFfYdH8Gipec8Eeeu0xXdbba9frFj0=OqFfea0dXdd9vqai=hGuQ8kuc9pgc9s8qqaq=dirpe0xb9q8qiLsFr0=vr0=vr0dc8meaabaqaciaacaGaaeqabaqabeGadaaakeaacqWGdbWqdaqadaqaaiabdkhaYjabcYcaSGGaciab=H7aXbGaayjkaiaawMcaaiabg2da9iabdoeadnaaBaaaleaacqaIWaamaeqaaOGaey4kaSIaem4zaCMaemOCaiNagi4yamMaei4Ba8Maei4CamNae8hUde3aaeWaaeaacqaIXaqmcqGHRaWkdaWcaaqaaiabdkfasnaaCaaaleqabaGaeG4mamdaaaGcbaGaeGOmaiJaemOCai3aaWbaaSqabeaacqaIZaWmaaaaaaGccaGLOaGaayzkaaGaeiilaWIaaCzcaiaaxMaadaqadaqaaiabikdaYaGaayjkaiaawMcaaaaa@4DEE@

where *C*_0 _is the concentration for position coordinates *θ *= *π*/2.

Now we proceed to find the cell trajectory in the vicinity of the obstacle. Substituting Eq.(2) in Eq.(l), after some simple algebraic manipulation we obtain:

drcdt=αgcos⁡θc(1−R3rc3),     (3)
 MathType@MTEF@5@5@+=feaafiart1ev1aaatCvAUfKttLearuWrP9MDH5MBPbIqV92AaeXatLxBI9gBaebbnrfifHhDYfgasaacH8akY=wiFfYdH8Gipec8Eeeu0xXdbba9frFj0=OqFfea0dXdd9vqai=hGuQ8kuc9pgc9s8qqaq=dirpe0xb9q8qiLsFr0=vr0=vr0dc8meaabaqaciaacaGaaeqabaqabeGadaaakeaadaWcaaqaaiabdsgaKjabdkhaYnaaBaaaleaacqWGJbWyaeqaaaGcbaGaemizaqMaemiDaqhaaiabg2da9GGaciab=f7aHjabdEgaNjGbcogaJjabc+gaVjabcohaZjab=H7aXnaaBaaaleaacqWGJbWyaeqaaOWaaeWaaeaacqaIXaqmcqGHsisldaWcaaqaaiabdkfasnaaCaaaleqabaGaeG4mamdaaaGcbaGaemOCai3aa0baaSqaaiabdogaJbqaaiabiodaZaaaaaaakiaawIcacaGLPaaacqGGSaalcaWLjaGaaCzcamaabmaabaGaeG4mamdacaGLOaGaayzkaaaaaa@4D74@

dθcdt=−αgsin⁡θcrc(1+R32rc3),     (4)
 MathType@MTEF@5@5@+=feaafiart1ev1aaatCvAUfKttLearuWrP9MDH5MBPbIqV92AaeXatLxBI9gBaebbnrfifHhDYfgasaacH8akY=wiFfYdH8Gipec8Eeeu0xXdbba9frFj0=OqFfea0dXdd9vqai=hGuQ8kuc9pgc9s8qqaq=dirpe0xb9q8qiLsFr0=vr0=vr0dc8meaabaqaciaacaGaaeqabaqabeGadaaakeaadaWcaaqaaiabdsgaKHGaciab=H7aXnaaBaaaleaacqWGJbWyaeqaaaGcbaGaemizaqMaemiDaqhaaiabg2da9iabgkHiTmaalaaabaGae8xSdeMaem4zaCMagi4CamNaeiyAaKMaeiOBa4Mae8hUde3aaSbaaSqaaiabdogaJbqabaaakeaacqWGYbGCdaWgaaWcbaGaem4yamgabeaaaaGcdaqadaqaaiabigdaXiabgUcaRmaalaaabaGaemOuai1aaWbaaSqabeaacqaIZaWmaaaakeaacqaIYaGmcqWGYbGCdaqhaaWcbaGaem4yamgabaGaeG4mamdaaaaaaOGaayjkaiaawMcaaiabcYcaSiaaxMaacaWLjaWaaeWaaeaacqaI0aanaiaawIcacaGLPaaaaaa@529A@

where *r*_*c *_and *θ*_*c *_are the position coordinates of the cell. Thus the equation of the cell's path is given by:

drcdθc=−rccot⁡θc(1−R3/rc3)(1+R3/2rc3),     (5)
 MathType@MTEF@5@5@+=feaafiart1ev1aaatCvAUfKttLearuWrP9MDH5MBPbIqV92AaeXatLxBI9gBaebbnrfifHhDYfgasaacH8akY=wiFfYdH8Gipec8Eeeu0xXdbba9frFj0=OqFfea0dXdd9vqai=hGuQ8kuc9pgc9s8qqaq=dirpe0xb9q8qiLsFr0=vr0=vr0dc8meaabaqaciaacaGaaeqabaqabeGadaaakeaadaWcaaqaaiabdsgaKjabdkhaYnaaBaaaleaacqWGJbWyaeqaaaGcbaGaemizaqgcciGae8hUde3aaSbaaSqaaiabdogaJbqabaaaaOGaeyypa0ZaaSaaaeaacqGHsislcqWGYbGCdaWgaaWcbaGaem4yamgabeaakiGbcogaJjabc+gaVjabcsha0jab=H7aXnaaBaaaleaacqWGJbWyaeqaaOWaaeWaaeaacqaIXaqmcqGHsislcqWGsbGudaahaaWcbeqaaiabiodaZaaakiabc+caViabdkhaYnaaDaaaleaacqWGJbWyaeaacqaIZaWmaaaakiaawIcacaGLPaaaaeaadaqadaqaaiabigdaXiabgUcaRiabdkfasnaaCaaaleqabaGaeG4mamdaaOGaei4la8IaeGOmaiJaemOCai3aa0baaSqaaiabdogaJbqaaiabiodaZaaaaOGaayjkaiaawMcaaaaacqGGSaalcaWLjaGaaCzcamaabmaabaGaeGynaudacaGLOaGaayzkaaaaaa@5C85@

which upon integrating gives:

(rcrc3−R3)1/2=sin⁡θcd.     (6)
 MathType@MTEF@5@5@+=feaafiart1ev1aaatCvAUfKttLearuWrP9MDH5MBPbIqV92AaeXatLxBI9gBaebbnrfifHhDYfgasaacH8akY=wiFfYdH8Gipec8Eeeu0xXdbba9frFj0=OqFfea0dXdd9vqai=hGuQ8kuc9pgc9s8qqaq=dirpe0xb9q8qiLsFr0=vr0=vr0dc8meaabaqaciaacaGaaeqabaqabeGadaaakeaadaqadaqaamaalaaabaGaemOCai3aaSbaaSqaaiabdogaJbqabaaakeaacqWGYbGCdaqhaaWcbaGaem4yamgabaGaeG4mamdaaOGaeyOeI0IaemOuai1aaWbaaSqabeaacqaIZaWmaaaaaaGccaGLOaGaayzkaaWaaWbaaSqabeaacqaIXaqmcqGGVaWlcqaIYaGmaaGccqGH9aqpdaWcaaqaaiGbcohaZjabcMgaPjabc6gaUHGaciab=H7aXnaaBaaaleaacqWGJbWyaeqaaaGcbaGaemizaqgaaiabc6caUiaaxMaacaWLjaWaaeWaaeaacqaI2aGnaiaawIcacaGLPaaaaaa@49E4@

**Figure 2:**
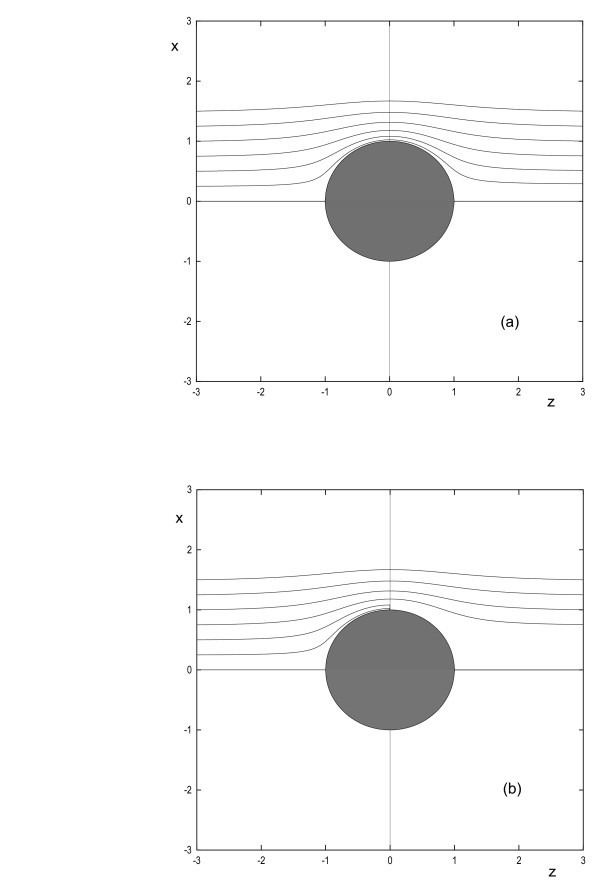
**Typical trajectories of the center of mass of a chemotactic cell with a non-absorbing object of unit radius placed in its path**. (a) The equation describing these paths is given by Eq. (6). Initially the cells are placed at *z *= -3 with *d *= 0.25, 0.5, 0.75,1,1.25,1.5. Note that here *d *corresponds to the *x *position of the cell at *z *= -3. In this diagram we do not consider any mechanical interaction between the cell and the sphere. (b) Same as (a) but now the cell radius is fixed at 0.1 and we allow interactions (i.e. attachment upon contact) between the cell and the sphere. Note that cells with *d *≲ 0.5 do NOT make it past the obstacle. This is because the capture radius, as given by Eq.(7), for a cell with radius 0.1 and an obstacle of unit radius is equal to 0.55.

This equation describes the spatial trajectory of the chemotactic cell. Note that *d *is the x position of the cell when it is still far away from the obstacle's influence. This is analogous to the impact parameter in the physics of scattering [18]. The trajectory is independent of the magnitude of the chemical gradient *g*, and of the chemotactic sensitivity *α*. Typical cell trajectories are illustrated in Fig. 2(a).

Thus we conclude that spatial perturbations of the chemical field in an object's vicinity, due to its physical presence, enable a chemotactic cell to avoid the obstacle simply by following the modified gradient. In many cases, there is NO need for an additional mechanism to sense and avoid the obstacle. This is not always the case since a cell can only directly avoid the obstacle if the distance of closest approach *r*_*min *_is greater than the sum of the obstacle's and the cell's radii, i.e. *r*_*min *_≥ *a *+ *R*.

A proper discussion of obstacle avoidance requires knowledge of the exact interaction between the cell and the obstacle upon mechanical contact. This is a subject of current research; generally, cells adhere to each other, to the extracellular matrix and to other biopolymers via various types of cell adhesion molecules. The strength of this adhesion depends sensitively on the specific type of cells and the obstacles under consideration. The dynamics of cell movement very close to the obstacle surface are also influenced by short-range hydrodynamic interactions between the two. In the interest of having an analytically tractable model, we shall ignore hydrodynamic interactions and assume irreversible adhesion of a cell to an obstacle upon mechanical contact. Our ensuing discussions regarding cell capture are based on this assumption. We note that since cells do not generally adhere permanently to obstacles, the estimates we shall derive for the probability of cell capture (which is a measure of the efficiency of chemotactic obstacle avoidance) are to be viewed as upper bounds for the real case. Further discussion of these assumptions is deferred to the last section of this article.

We shall now quantify the efficiency of chemotactic obstacle avoidance. A convenient quantity to calculate is the capture radius *r*_*cap*_. For a cell of radius *a *moving in a straight line trajectory towards a spherical obstacle of radius *R*, the capture radius is *r*_*cap *_= *a *+ *R*. However, the streamline-like trajectories induced by spatial perturbations in the chemical field imply that *r*_*cap *_<* a *+ *R*. To calculate the actual capture radius, consider the following argument. From Eq. (5) it can be seen that the closest distance of approach *r*_*min *_occurs at *θ *= *π*/2; the capture radius *r*_*cap *_is then given by the value of *d *for which *r*_*min *_= *a *+ *R*. From Eq.(6), we then have:

rcap=R(1+δ)3−11+δ,     (7)
 MathType@MTEF@5@5@+=feaafiart1ev1aaatCvAUfKttLearuWrP9MDH5MBPbIqV92AaeXatLxBI9gBaebbnrfifHhDYfgasaacH8akY=wiFfYdH8Gipec8Eeeu0xXdbba9frFj0=OqFfea0dXdd9vqai=hGuQ8kuc9pgc9s8qqaq=dirpe0xb9q8qiLsFr0=vr0=vr0dc8meaabaqaciaacaGaaeqabaqabeGadaaakeaacqWGYbGCdaWgaaWcbaGaem4yamMaemyyaeMaemiCaahabeaakiabg2da9iabdkfasnaakaaabaWaaSaaaeaadaqadaqaaiabigdaXiabgUcaRGGaciab=r7aKbGaayjkaiaawMcaamaaCaaaleqabaGaeG4mamdaaOGaeyOeI0IaeGymaedabaGaeGymaeJaey4kaSIae8hTdqgaaaWcbeaakiabcYcaSiaaxMaacaWLjaWaaeWaaeaacqaI3aWnaiaawIcacaGLPaaaaaa@44E4@

where *δ *= *a*/*R*. The physical relevance of the capture radius can be appreciated by the following simple experiment, which is illustrated in Fig. 2(b). Suppose that at time *t *= 0, a cell is randomly positioned on a circle in the x-y plane with center (*x *= 0, *y *= 0, *z *= -3) and radius *R*'. We repeat this experiment a large number of times, each time noting whether the cell is eventually captured. We would observe that cells that were initially within a radius *d *= *r*_*cap *_are captured by the obstacle; cells initially within the annulus defined by the radius range *r*_*cap *_<*d *<*R*' would, however, chemotactically avoid the obstacle. Another measure of the efficiency of chemotactic obstacle avoidance is as follows. Consider again the experiment depicted in Fig. 2b. with the difference that *R*' = *a *+ *R*. What is the probability that the cells will be able to avoid the obstacle? In general, this quantity is simply given by the expression: *P*_*cap *_= *π*rcap2
 MathType@MTEF@5@5@+=feaafiart1ev1aaatCvAUfKttLearuWrP9MDH5MBPbIqV92AaeXatLxBI9gBaebbnrfifHhDYfgasaacH8akY=wiFfYdH8Gipec8Eeeu0xXdbba9frFj0=OqFfea0dXdd9vqai=hGuQ8kuc9pgc9s8qqaq=dirpe0xb9q8qiLsFr0=vr0=vr0dc8meaabaqaciaacaGaaeqabaqabeGadaaakeaacqWGYbGCdaqhaaWcbaGaem4yamMaemyyaeMaemiCaahabaGaeGOmaidaaaaa@333B@/*π *(*a *+ *R*)^2^. Note that if we had to ignore the spatial perturbation of the field, then *P*_*cap *_= 1. Otherwise, we have:

Pcap=(1+δ)3−1(1+δ)3.     (8)
 MathType@MTEF@5@5@+=feaafiart1ev1aaatCvAUfKttLearuWrP9MDH5MBPbIqV92AaeXatLxBI9gBaebbnrfifHhDYfgasaacH8akY=wiFfYdH8Gipec8Eeeu0xXdbba9frFj0=OqFfea0dXdd9vqai=hGuQ8kuc9pgc9s8qqaq=dirpe0xb9q8qiLsFr0=vr0=vr0dc8meaabaqaciaacaGaaeqabaqabeGadaaakeaacqWGqbaudaWgaaWcbaGaem4yamMaemyyaeMaemiCaahabeaakiabg2da9maalaaabaWaaeWaaeaacqaIXaqmcqGHRaWkiiGacqWF0oazaiaawIcacaGLPaaadaahaaWcbeqaaiabiodaZaaakiabgkHiTiabigdaXaqaamaabmaabaGaeGymaeJaey4kaSIae8hTdqgacaGLOaGaayzkaaWaaWbaaSqabeaacqaIZaWmaaaaaOGaeiOla4IaaCzcaiaaxMaadaqadaqaaiabiIda4aGaayjkaiaawMcaaaaa@4608@

Thus *P*_*cap *_< 1 and it decreases monotonically as a function of *δ *= *a*/*R*. For cells with radius *a *≳ *R*/4, the capture probability is greater than 0.5, so obstacle avoidance by simply following chemotactic gradients is not efficient for cells larger than this. We note that the total capture probability should actually be calculated in the limit *R*' → ∞. However, since cells initially within an annulus defined by the radius range *d *> *a *+ *R *always avoid the obstacle, *P*_*cap *_given by Eq. (8) has to be equal to *P*_*cap *_calculated in the limit of infinitely large *R*'.

We now investigate the apparent geometric similarity of the chemotactic cell trajectories around a non-absorbing obstacle (Fig. 2) to the streamlines of an incompressible and inviscid fluid around a spherical object. Consider the irrotational flow of an incompressible and inviscid fluid past a spherical object [19]. If **u **is the fluid velocity, then irrotational flow implies that ∇ × **u **= 0; this can also be expressed in terms of a scalar function, *φ*, as **u **= ∇ *φ*. Furthermore, incompressibility implies ∇·**u **= 0. Combining the irrotational and incompressibility conditions, we obtain Laplace's equation ∇^2 ^*φ *= 0. *φ *is thus commonly referred to as the velocity potential. Since the normal component of the fluid velocity **u **has to be zero at the obstacle's surface, we have the boundary condition **n·**∇ *φ *= 0. The movement of a chemotactic cell in an external uniform chemical field perturbed by an obstacle is thus mathematically analogous: **u **≡ x˙
 MathType@MTEF@5@5@+=feaafiart1ev1aaatCvAUfKttLearuWrP9MDH5MBPbIqV92AaeXatLxBI9gBaebbnrfifHhDYfgasaacH8akY=wiFfYdH8Gipec8Eeeu0xXdbba9frFj0=OqFfea0dXdd9vqai=hGuQ8kuc9pgc9s8qqaq=dirpe0xb9q8qiLsFr0=vr0=vr0dc8meaabaqaciaacaGaaeqabaqabeGadaaakeaaieqacuWF4baEgaGaaaaa@2E34@_*c *_and *φ *≡ *C*. The mathematical form of the chemotactic cell trajectories is therefore exactly the same as that describing streamlines of fluid flow around an obstacle. This has one important implication: It is not possible to distinguish between the case of a chemotactic cell following a gradient around an obstacle in a stationary fluid and a non-chemotactic cell dragged past a stationary obstacle by a moving fluid. This equivalence is strictly speaking only valid for the case of a chemotactic cell with velocity directly proportional to the chemical gradient. As previously mentioned, this assumption is correct if the absolute value of the chemical concentration is small. More generally, the chemotactic velocity is a non-linear function of the chemical gradient and the chemical concentration, examples being a logarithmic response due to sensory adaptation (see [20] and references therein) and more complicated responses [9]. The equivalence may also be broken by temporal delays between changes in the chemical stimulus and the ensuing chemotactic response. As we shall see in the next section, it also breaks down if the obstacle absorbs some of the chemotactic chemical at its surface.

### The absorbing obstacle case

In this subsection we explore the effect of the obstacle's absorption properties on the cell trajectories and the capture probability. In all our previous discussions we have assumed that the obstacle does not absorb any chemical. However, in a number of cases the chemotactic chemical might take part in reactions on the obstacle's surface, meaning that some of the molecules will be sequestered upon reaching the surface. In this section we consider the opposite case to that in the previous section: the obstacle is assumed to be a perfect sink for the chemical, sequestering every molecule that reaches its surface. The chemical field around such an obstacle is obtained by solving Laplace's equation ∇^2 ^*C *(*r*, *θ*, *φ*) = 0 with boundary conditions: ∇ **C **= *g *z^
 MathType@MTEF@5@5@+=feaafiart1ev1aaatCvAUfKttLearuWrP9MDH5MBPbIqV92AaeXatLxBI9gBaebbnrfifHhDYfgasaacH8akY=wiFfYdH8Gipec8Eeeu0xXdbba9frFj0=OqFfea0dXdd9vqai=hGuQ8kuc9pgc9s8qqaq=dirpe0xb9q8qiLsFr0=vr0=vr0dc8meaabaqaciaacaGaaeqabaqabeGadaaakeaacuWG6bGEgaqcaaaa@2E39@ in the limit *r *→ ∞ and *C *(*r *= *R*) = 0. The chemical field is then described by an equation of the form:

C(r,θ)=C0(1−Rr)+grcos⁡θ(1−R3r3),     (9)
 MathType@MTEF@5@5@+=feaafiart1ev1aaatCvAUfKttLearuWrP9MDH5MBPbIqV92AaeXatLxBI9gBaebbnrfifHhDYfgasaacH8akY=wiFfYdH8Gipec8Eeeu0xXdbba9frFj0=OqFfea0dXdd9vqai=hGuQ8kuc9pgc9s8qqaq=dirpe0xb9q8qiLsFr0=vr0=vr0dc8meaabaqaciaacaGaaeqabaqabeGadaaakeaacqWGdbWqdaqadaqaaiabdkhaYjabcYcaSGGaciab=H7aXbGaayjkaiaawMcaaiabg2da9iabdoeadnaaBaaaleaacqaIWaamaeqaaOWaaeWaaeaacqaIXaqmcqGHsisldaWcaaqaaiabdkfasbqaaiabdkhaYbaaaiaawIcacaGLPaaacqGHRaWkcqWGNbWzcqWGYbGCcyGGJbWycqGGVbWBcqGGZbWCcqWF4oqCdaqadaqaaiabigdaXiabgkHiTmaalaaabaGaemOuai1aaWbaaSqabeaacqaIZaWmaaaakeaacqWGYbGCdaahaaWcbeqaaiabiodaZaaaaaaakiaawIcacaGLPaaacqGGSaalcaWLjaGaaCzcamaabmaabaGaeGyoaKdacaGLOaGaayzkaaaaaa@5325@

where *C*_0 _is the concentration for position coordinates (*r *→ ∞, *θ *= *π*/2). As in the previous subsection, we obtain the equations for *dr*_*c*_/*dt *and *dθ*_*c*_/*dt *and divide to obtain:

drcdθc=−C0R/rc3+αgrccos⁡θc(1+2R3/rc3)αgsin⁡θc(1−R3/rc3).     (10)
 MathType@MTEF@5@5@+=feaafiart1ev1aaatCvAUfKttLearuWrP9MDH5MBPbIqV92AaeXatLxBI9gBaebbnrfifHhDYfgasaacH8akY=wiFfYdH8Gipec8Eeeu0xXdbba9frFj0=OqFfea0dXdd9vqai=hGuQ8kuc9pgc9s8qqaq=dirpe0xb9q8qiLsFr0=vr0=vr0dc8meaabaqaciaacaGaaeqabaqabeGadaaakeaadaWcaaqaaiabdsgaKjabdkhaYnaaBaaaleaacqWGJbWyaeqaaaGcbaGaemizaqgcciGae8hUde3aaSbaaSqaaiabdogaJbqabaaaaOGaeyypa0JaeyOeI0YaaSaaaeaacqWGdbWqdaWgaaWcbaGaeGimaadabeaakiabdkfasjabc+caViabdkhaYnaaDaaaleaacqWGJbWyaeaacqaIZaWmaaGccqGHRaWkcqWFXoqycqWGNbWzcqWGYbGCdaWgaaWcbaGaem4yamgabeaakiGbcogaJjabc+gaVjabcohaZjab=H7aXnaaBaaaleaacqWGJbWyaeqaaOWaaeWaaeaacqaIXaqmcqGHRaWkcqaIYaGmcqWGsbGudaahaaWcbeqaaiabiodaZaaakiabc+caViabdkhaYnaaDaaaleaacqWGJbWyaeaacqaIZaWmaaaakiaawIcacaGLPaaaaeaacqWFXoqycqWGNbWzcyGGZbWCcqGGPbqAcqGGUbGBcqWF4oqCdaWgaaWcbaGaem4yamgabeaakmaabmaabaGaeGymaeJaeyOeI0IaemOuai1aaWbaaSqabeaacqaIZaWmaaGccqGGVaWlcqWGYbGCdaqhaaWcbaGaem4yamgabaGaeG4mamdaaaGccaGLOaGaayzkaaaaaiabc6caUiaaxMaacaWLjaWaaeWaaeaacqaIXaqmcqaIWaamaiaawIcacaGLPaaaaaa@73C2@

**Figure 3:**
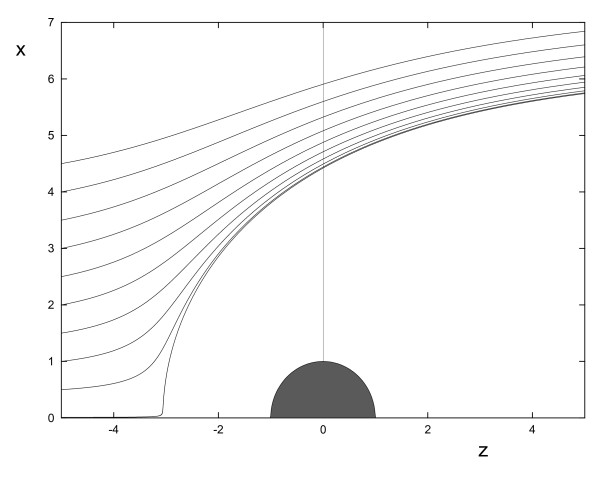
**Typical trajectories of the center of mass of a chemotactic cell with a perfectly absorbing object of unit radius placed in its path**. All parameters with the exception of *C*_0 _are set to unity. The value of *C*_0 _is equal to 10. The equation describing these paths is given by Eq. (11). Initially the cells are placed at *z *= -5 with *d *= 0.001, 0.5 – 4.5 in 0.5 step intervals. In this diagram we do not consider any mechanical interaction between the cell and the sphere.

Direct solution of this equation is a non-trivial task. A more straightforward approach involves using Stoke's stream function, *ψ*, which is a common method for solving hydrodynamic problems [19]. The trajectory of a cell corresponds to *ψ *= *k*, where the constant *k *is determined by the cell's position when it is far away from the obstacle. The stream function in a plane is determined from the equations: *∂ ψ*/*∂r *= - sin *θ ∂C*/*∂θ *and *∂ψ*/*∂θ *= *r*^2 ^sin *θ ∂C*/*∂r*. Solving these simple equations for *ψ*, equating this resultant expression to *k *(this is determined by assuming that the initial position of the cell is (*x, z*) = (*d, e*)) and substituting *r *= *r*_*c *_and *θ *= *θ*_*c*_, we obtain the final equation for the trajectory of the cell:

rc2sin⁡2θc(1+2R3rc3)−2C0Rcos⁡θcαg=d2−2C0Reαgd2+e2,     (11)
 MathType@MTEF@5@5@+=feaafiart1ev1aaatCvAUfKttLearuWrP9MDH5MBPbIqV92AaeXatLxBI9gBaebbnrfifHhDYfgasaacH8akY=wiFfYdH8Gipec8Eeeu0xXdbba9frFj0=OqFfea0dXdd9vqai=hGuQ8kuc9pgc9s8qqaq=dirpe0xb9q8qiLsFr0=vr0=vr0dc8meaabaqaciaacaGaaeqabaqabeGadaaakeaacqWGYbGCdaqhaaWcbaGaem4yamgabaGaeGOmaidaaOGagi4CamNaeiyAaKMaeiOBa42aaWbaaSqabeaacqaIYaGmaaacciGccqWF4oqCdaWgaaWcbaGaem4yamgabeaakmaabmaabaGaeGymaeJaey4kaSYaaSaaaeaacqaIYaGmcqWGsbGudaahaaWcbeqaaiabiodaZaaaaOqaaiabdkhaYnaaDaaaleaacqWGJbWyaeaacqaIZaWmaaaaaaGccaGLOaGaayzkaaGaeyOeI0YaaSaaaeaacqaIYaGmcqWGdbWqdaWgaaWcbaGaeGimaadabeaakiabdkfasjGbcogaJjabc+gaVjabcohaZjab=H7aXnaaBaaaleaacqWGJbWyaeqaaaGcbaGae8xSdeMaem4zaCgaaiabg2da9iabdsgaKnaaCaaaleqabaGaeGOmaidaaOGaeyOeI0YaaSaaaeaacqaIYaGmcqWGdbWqdaWgaaWcbaGaeGimaadabeaaieGakiab+jfasjab+vgaLbqaaiab=f7aHjabdEgaNnaakaaabaGaemizaq2aaWbaaSqabeaacqaIYaGmaaGccqGHRaWkcqWGLbqzdaahaaWcbeqaaiabikdaYaaaaeqaaaaakiabcYcaSiaaxMaacaWLjaWaaeWaaeaacqaIXaqmcqaIXaqmaiaawIcacaGLPaaaaaa@6BE9@

which satisfies Eq.(10), as can be verified by direct substitution. Contrary to the case in which the obstacle does not absorb any chemical, we notice in this case that the geometrical form of the cell path depends on the magnitude of the chemical gradient *g*, the chemotactic sensitivity *α*, the absolute value of the chemical concentration *C*_0 _and the initial distance of the cell along the z-axis *e*. Typical cell trajectories are illustrated in Fig. 3. Note the considerable difference from the cell trajectories typical of the non-absorbing case (see Fig. 2a).

Several observations can be made: (i) the trajectories are not symmetrical about the obstacle, i.e. when it passes the obstacle, a cell suffers a permanent change in its trajectory; (ii) through-out its motion past the obstacle, a cell never comes very close, even when *d *is very small; (iii) the effect of the obstacle on the cell's movement is appreciable even at large distances *r *>> *R*.

**Figure 4:**
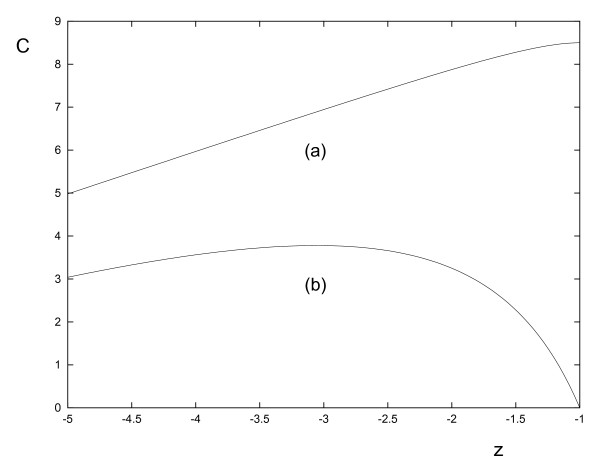
**Graph of the concentration C versus distance z on the line *x *= *y *= 0**. for (a) a non-absorbing obstacle (b) an absorbing obstacle. The parameter values are all set to unity with the exception of *C*_0_, which has value 10. Note that the obstacle has its center at the origin and thus a boundary at *z *= -1.

These observations can all be explained by considering the obstacle-perturbed field Eq.(9). Consider a cell that is initially placed very close to the z-axis, i.e. *d *is very small. The force it experiences in the z-direction is proportional to the concentration gradient in this direction, a graph of which is shown in Fig. 4(b). The cell initially approaches the obstacle's surface but stops moving towards it when the force becomes zero. In the region close to the obstacle's surface, the gradient is negative and thus this region is inaccessible to the cell. The gradient is negative because at the obstacle's surface the concentration is zero, a condition dictated by the obstacle being an idealized sink. Note that this was not the case when the obstacle was non-absorbent, in which case the gradient was always positive and became zero only at the surface (see Fig. 4(a)). This explains observation (ii) above.

We now use this argument to calculate the minimum radius required for a cell to be captured and hence deduce the corresponding capture probability. Note that the latter quantity refers to the experiment introduced in the previous section. The cells passing the closest to the obstacle are the ones initially close to the z-axis, i.e. *d *is small. For such cells the distance of closest approach *r*_*min *_occurs at *θ *≃ *π*. This can be most easily demonstrated by substituting Eq. (11) with *d *= 0 in Eq. (10) with the R.H.S equal to zero; solving for *θ *gives the angle at which the cell approaches the obstacle most closely. Thus for cells with small *d*, the distance of closest approach, *r*_*min*_, is given by the z position (along the line *x *= *y *= 0) at which the gradient in the z-direction becomes zero, which satisfies the equation:

rmin3−C0Rgrmin+2R3g=0.     (12)
 MathType@MTEF@5@5@+=feaafiart1ev1aaatCvAUfKttLearuWrP9MDH5MBPbIqV92AaeXatLxBI9gBaebbnrfifHhDYfgasaacH8akY=wiFfYdH8Gipec8Eeeu0xXdbba9frFj0=OqFfea0dXdd9vqai=hGuQ8kuc9pgc9s8qqaq=dirpe0xb9q8qiLsFr0=vr0=vr0dc8meaabaqaciaacaGaaeqabaqabeGadaaakeaacqWGYbGCdaqhaaWcbaacbiGae8xBa0Mae8xAaKMae8NBa4gabaGaeG4mamdaaOGaeyOeI0YaaSaaaeaacqWGdbWqdaWgaaWcbaGaeGimaadabeaakiabdkfasbqaaiabdEgaNbaacqWGYbGCdaWgaaWcbaGae8xBa0Mae8xAaKMae8NBa4gabeaakiabgUcaRmaalaaabaGaeGOmaiJaemOuai1aaWbaaSqabeaacqaIZaWmaaaakeaacqWGNbWzaaGaeyypa0JaeGimaaJaeiOla4IaaCzcaiaaxMaadaqadaqaaiabigdaXiabikdaYaGaayjkaiaawMcaaaaa@4BEE@

**Figure 5:**
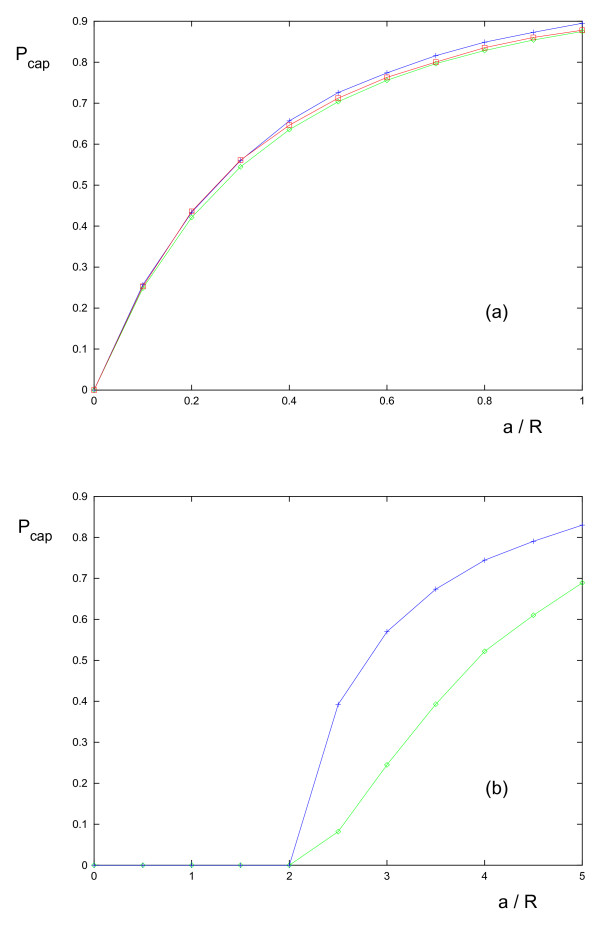
**Graph of the capture probability *P*_*cap *_versus the ratio of the cell to obstacle radius *a/R***. for (a) a non-absorbing obstacle (b) a perfectly absorbing obstacle. The parameter values are all set to unity with the exception of *C*_0_, which has value 10. Notice that for the second case only cells larger than a certain critical size are captured. The data for these plots were obtained from theory (green) and simulations (blue, red) for (a). The blue curve is computed using the gradient in the middle of the cell and the red curve is computed using the gradient across its body. The data for (b) are from simulations only, with the green curve representing data with the central gradient and the blue curve representing data using the gradient across the cell's body.

Then a cell is captured if its radius *a *satisfies the condition *a *+ *R *≥ *r*_*min*_. Cells with a radius smaller than the critical radius *a *= *r*_*min *_- *R *will not be captured, irrespective of *d*. For cells larger than the critical radius, capture may occur if *d *is small enough, but not in general. Thus the capture probability is zero for cells smaller than the critical radius and non-zero otherwise. This differs from the case of a non-absorbing obstacle, in which the capture probability is always greater than zero irrespective of cell size (see Fig. 5). For the parameter values used in Fig. 5, the above equation predicts *r*_*min *_= 3.06, which implies *a *≥ 2.06 for capture, a fact verified by the simulation data in the figure. Note also that the simulations (see Fig. 5) indicate that the theoretical results for both the absorbing and the non-absorbing obstacle cases, which were derived on the basis of a cell sensing the gradient in its center, are also qualitatively reproduced if cells sense the gradient across their bodies.

Observation (iii) can be explained by noting that the perturbation in the chemical field, Eq.(9), decays much more slowly for long distances (decays as 1/*r*) than it does for the non-absorbing case (decays as 1/*r*^3^). Observation (i) is explained by the fact that after a cell passes the obstacle it does not experience a force pulling it back towards the obstacle; this is because the chemical gradient in the x-direction at any point in space always points away from the obstacle, since the concentration at the surface is zero.

### The effect of noise on the capture probability

In this section we study the effect of noise on the obstacle avoiding abilities of chemotactic cells. In the deterministic case, the cell's motion was completely determined by the local chemical gradient. We now relax this condition by requiring that the cell's motion is partly determined by intrinsic noise and partly by the gradient. The cell's motion will be modeled as a random walk, characterized by a cell diffusion coefficient *D*, biased in the direction of increasing gradient.

The stochastic description is in all aspects similar to the deterministic one, with the exception that the equation describing the cell's motion has an extra noise term:

x˙
 MathType@MTEF@5@5@+=feaafiart1ev1aaatCvAUfKttLearuWrP9MDH5MBPbIqV92AaeXatLxBI9gBaebbnrfifHhDYfgasaacH8akY=wiFfYdH8Gipec8Eeeu0xXdbba9frFj0=OqFfea0dXdd9vqai=hGuQ8kuc9pgc9s8qqaq=dirpe0xb9q8qiLsFr0=vr0=vr0dc8meaabaqaciaacaGaaeqabaqabeGadaaakeaaieqacuWF4baEgaGaaaaa@2E34@_*c *_(*t*) = *α ∇C *(**x**_*c *_(*t*), *t*) + *ξ *(*t*).     (13)

This is a Langevin equation [21]. The stochastic variable *ξ *is white noise defined through the relations: ⟨*ξ*_*a *_(*t*)⟩ = 0 and ⟨*ξ*_*a *_(*t*) *ξ*_*b *_(*t*')⟩ = 2 *D δ*_*a,b*_*δ *(*t *- *t*'), where *a *and *b *refer to the spatial components of the noise vectors and *D *is the cell's diffusion coefficient. Note that the angled brackets denote the statistical average. For convenience, the cartesian components of the noise vector will be denoted as *ξ *(*t*) = (*ξ*_*x *_(*t*), *ξ*_*y *_(*t*), *ξ*_*z *_(*t*)). Assuming that the obstacle is non-absorbing, the concentration field *C *is as given by Eq.(2). As before, we switch to a description in spherical polar coordinates. The equations of motion for the chemotactic cell are then:

drcdt=αgcos⁡θc(1+R3rc3)+γ(θc,φc,t),     (14)
 MathType@MTEF@5@5@+=feaafiart1ev1aaatCvAUfKttLearuWrP9MDH5MBPbIqV92AaeXatLxBI9gBaebbnrfifHhDYfgasaacH8akY=wiFfYdH8Gipec8Eeeu0xXdbba9frFj0=OqFfea0dXdd9vqai=hGuQ8kuc9pgc9s8qqaq=dirpe0xb9q8qiLsFr0=vr0=vr0dc8meaabaqaciaacaGaaeqabaqabeGadaaakeaadaWcaaqaaiabdsgaKjabdkhaYnaaBaaaleaacqWGJbWyaeqaaaGcbaGaemizaqMaemiDaqhaaiabg2da9GGaciab=f7aHjabdEgaNjGbcogaJjabc+gaVjabcohaZjab=H7aXnaaBaaaleaacqWGJbWyaeqaaOWaaeWaaeaacqaIXaqmcqGHRaWkdaWcaaqaaiabdkfasnaaCaaaleqabaGaeG4mamdaaaGcbaGaemOCai3aa0baaSqaaiabdogaJbqaaiabiodaZaaaaaaakiaawIcacaGLPaaacqGHRaWkcqWFZoWzdaqadaqaaiab=H7aXnaaBaaaleaacqWGJbWyaeqaaOGaeiilaWIae8NXdy2aaSbaaSqaaiabdogaJbqabaGccqGGSaalcqWG0baDaiaawIcacaGLPaaacqGGSaalcaWLjaGaaCzcamaabmaabaGaeGymaeJaeGinaqdacaGLOaGaayzkaaaaaa@5C08@

dθcdt=−αgsin⁡θcrc(1+R32rc3)+cos⁡θcγ(θc,φc,t)−ξz(t)rcsin⁡θc,     (15)
 MathType@MTEF@5@5@+=feaafiart1ev1aaatCvAUfKttLearuWrP9MDH5MBPbIqV92AaeXatLxBI9gBaebbnrfifHhDYfgasaacH8akY=wiFfYdH8Gipec8Eeeu0xXdbba9frFj0=OqFfea0dXdd9vqai=hGuQ8kuc9pgc9s8qqaq=dirpe0xb9q8qiLsFr0=vr0=vr0dc8meaabaqaciaacaGaaeqabaqabeGadaaakeaadaWcaaqaaiabdsgaKHGaciab=H7aXnaaBaaaleaacqWGJbWyaeqaaaGcbaGaemizaqMaemiDaqhaaiabg2da9iabgkHiTmaalaaabaGae8xSdeMaem4zaCMagi4CamNaeiyAaKMaeiOBa4Mae8hUde3aaSbaaSqaaiabdogaJbqabaaakeaacqWGYbGCdaWgaaWcbaGaem4yamgabeaaaaGcdaqadaqaaiabigdaXiabgUcaRmaalaaabaGaemOuai1aaWbaaSqabeaacqaIZaWmaaaakeaacqaIYaGmcqWGYbGCdaqhaaWcbaGaem4yamgabaGaeG4mamdaaaaaaOGaayjkaiaawMcaaiabgUcaRmaalaaabaGagi4yamMaei4Ba8Maei4CamNae8hUde3aaSbaaSqaaiabdogaJbqabaGccqaHZoWzdaqadaqaaiab=H7aXnaaBaaaleaacqWGJbWyaeqaaOGaeiilaWIae8NXdy2aaSbaaSqaaiabdogaJbqabaGccqGGSaalcqWG0baDaiaawIcacaGLPaaacqGHsislcqWF+oaEdaWgaaWcbaGaemOEaOhabeaakmaabmaabaGaemiDaqhacaGLOaGaayzkaaaabaGaemOCai3aaSbaaSqaaiabdogaJbqabaGccyGGZbWCcqGGPbqAcqGGUbGBcqWF4oqCdaWgaaWcbaGaem4yamgabeaaaaGccqGGSaalcaWLjaGaaCzcamaabmaabaGaeGymaeJaeGynaudacaGLOaGaayzkaaaaaa@7A56@

dφcdt=cos⁡θccot⁡θcrccos⁡φc(ξy(t)−ξx(t)cot⁡φc),     (16)
 MathType@MTEF@5@5@+=feaafiart1ev1aaatCvAUfKttLearuWrP9MDH5MBPbIqV92AaeXatLxBI9gBaebbnrfifHhDYfgasaacH8akY=wiFfYdH8Gipec8Eeeu0xXdbba9frFj0=OqFfea0dXdd9vqai=hGuQ8kuc9pgc9s8qqaq=dirpe0xb9q8qiLsFr0=vr0=vr0dc8meaabaqaciaacaGaaeqabaqabeGadaaakeaadaWcaaqaaiabdsgaKHGaciab=z8aMnaaBaaaleaacqWGJbWyaeqaaaGcbaGaemizaqMaemiDaqhaaiabg2da9maalaaabaGagi4yamMaei4Ba8Maei4CamNae8hUde3aaSbaaSqaaiabdogaJbqabaGccyGGJbWycqGGVbWBcqGG0baDcqWF4oqCdaWgaaWcbaGaem4yamgabeaaaOqaaiabdkhaYnaaBaaaleaacqWGJbWyaeqaaOGagi4yamMaei4Ba8Maei4CamNae8NXdy2aaSbaaSqaaiabdogaJbqabaaaaOWaaeWaaeaacqWF+oaEdaWgaaWcbaGaemyEaKhabeaakmaabmaabaGaemiDaqhacaGLOaGaayzkaaGaeyOeI0YaaSaaaeaacqWF+oaEdaWgaaWcbaGaemiEaGhabeaakmaabmaabaGaemiDaqhacaGLOaGaayzkaaaabaGagi4yamMaei4Ba8MaeiiDaqNae8NXdy2aaSbaaSqaaiabdogaJbqabaaaaaGccaGLOaGaayzkaaGaeiilaWIaaCzcaiaaxMaadaqadaqaaiabigdaXiabiAda2aGaayjkaiaawMcaaaaa@6A7B@

where

*γ *(*θ*_*c*_, *φ*_*c*_, *t*) = sin *θ*_*c *_cos *φ*_*c *_*ξ*_*x *_(*t*) + sin *θ*_*c *_sin *φ*_*c *_*ξ*_*y *_(*t*) + cos *θ*_*c *_*ξ*_*z *_(*t*).     (17)

In contrast to the deterministic case, the cell's movement is not restricted to a plane and is dependent on the magnitude of the chemical gradient *g*. Note that we recover the deterministic case by setting the noise to zero, which implies *θ*_*c *_= *constant *(motion in a plane) and independence of the cell's trajectory from the gradient (this follows by dividing Eq. (14) by Eq. (15) as done in the previous section). A standard general method for analyzing stochastic differential equations involves a small noise expansion [21] about the deterministic solution. This method rests on the assumption that a deterministic explicit solution is known, i.e. *r*_*c *_(*t*) = *f *(*t*), *θ*_*c *_(*t*) = *g *(*t*), *φ*_*c *_(*t*) = *h *(*t*). No such explicit solutions can be obtained in our case; this can most easily be seen by using Eq.(6) to derive an expression for cos *θ*_*c*_, which is then substituted in Eq.(3) to obtain a first-order non-linear differential equation for *r*_*c *_(*t*). Hence the above equations do not lend themselves easily to analysis; it is not generally possible to derive equations for the trajectory, capture radius and capture probability for the stochastic case. Thus our investigation of the role and effect of noise on the dynamics will be solely through numerical simulation.

**Figure 6:**
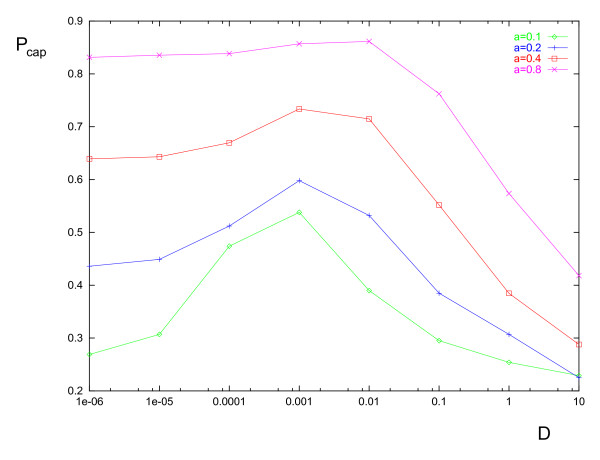
**Graph showing the variation of the capture probability *P*_*cap *_with the cell diffusion coefficient *D***. The variation is shown for different values of the cell radius *a*. The obstacle is non-absorbing and has unit radius. The parameter values are all set to unity.

We probe the system's stochastic behavior by measuring the capture probability *P*_*cap *_as a function of the cell diffusion coefficient *D*, which is a measure of the noise strength. To measure the capture probability the following setup is used. A spherical obstacle of radius *R *= 1 is placed at the origin as in Fig. 1. At *t *= 0, a cell of radius *a *is randomly placed on a circle in the x-y plane of radius *a *+ *R *and center coordinates *x *= 0; *y *= 0; *z *= -3. The cell motion is determined by numerically integrating Eq. (13). At each time step, the algorithm computes the new cell position and checks whether the cell has come into contact with the obstacle. If this condition is found to be true then the simulation stops and a counter is increased by one. If the condition is false then the program keeps running until either the condition becomes true or the cell reaches the plane *z *= 3. Note that the counter is not reset to zero after the program finishes. This simple program is run 5 × 10^4 ^times; the capture probability is then given by the value of the counter divided by 5 × 10^4^. Note that stopping the simulation when the plane *z *= 3 is reached is an arbitrary choice, initially made to mirror the initial position symmetrically; we found that changing the stopping value of *z *generally has minor effects on *P*_*cap *_except when the cell diffusion coefficient is substantially large. This is because in the latter case the cell has a significant probability of being captured after passing the obstacle (by moving against the gradient), which does not happen at small diffusion coefficients. The larger the stopping value of *z*, the higher the probability that this will occur. The results of our simulations are shown in Fig. 6. Two general observations can be made: (i) For any given *D*, the capture probability is proportional to the cell radius. This is expected, (ii) *P*_*cap *_peaks at a particular value of *D*. This peak behavior is clearly distinguishable and relevant only for small values of the cell radius, *a *≲ 0.4.

This last observation requires some explanation. The peak in the capture probability separates two distinct regimes of dynamical behavior: (i) the chemotaxis-dominated regime in which cells strongly follow the chemical gradient (ii) the diffusion-dominated regime in which the cell behavior is mostly stochastic and only weakly determined by the chemotactic gradients. The two regimes are approximately determined by the two timescales: *τ*_*c*_~*L*/*αg *and *τ*_*d*_~*L*^2^/6 *D*, where *L *= 2 *R *is the obstacle's diameter. Cell movement is mainly by chemotaxis when *τ*_*c *_≪ *τ*_*d *_(chemotaxis-dominated regime) and principally by diffusion when *τ*_*d *_≪*τ*_*c *_(diffusion-dominated regime). This is indeed conceptually parallel to the advection-dominated (high Peclet number) and diffusion-dominated (low Peclet number) regimes in models of chemical transport in fluids. Whereas the cell's x-position is approximately limited to the range *x *∈ [-(*a *+ *R*), *a *+ *R*] for very small diffusion, the range is much greater for large diffusion. Of course the larger the range, the smaller the probability of the cell being captured. The range is dictated by the magnitude of the fluctuations in the cell's position, which grows roughly as D
 MathType@MTEF@5@5@+=feaafiart1ev1aaatCvAUfKttLearuWrP9MDH5MBPbIqV92AaeXatLxBI9gBaebbnrfifHhDYfgasaacH8akY=wiFfYdH8Gipec8Eeeu0xXdbba9frFj0=OqFfea0dXdd9vqai=hGuQ8kuc9pgc9s8qqaq=dirpe0xb9q8qiLsFr0=vr0=vr0dc8meaabaqaciaacaGaaeqabaqabeGadaaakeaadaGcaaqaaiabdseaebWcbeaaaaa@2DD8@; hence in the diffusion-dominated regime we expect the probability of capture to decrease with increasing diffusion coefficient.

What remains to be explained is the increase in capture probability with noise in the chemotaxis-dominated regime. Here, a cell roughly follows the trajectories of the deterministic case. Consider two different and non-interacting cells: cell 1 is placed just inside the capture radius *d *= *r*_*cap *_- *δ x *and cell 2 just outside of the capture radius *d *= *r*_*cap *_+ *δ x*. Capture, if it occurs, will happen at or near the obstacle's equator (*θ *= *π*/2) since this is the distance of closest approach. Owing to noise fluctuations, cells 1 and 2 may switch positions in the course of their path towards the obstacle. If it was equally probable for the cells to switch positions, then the capture probability would not change from the deterministic case. However, this is not the case: cell 1 in the course of its path towards the obstacle's equator passes closer to the obstacle's surface than cell 2, implying that the probability of cell 1 leaving the capture volume is less than the probability of cell 2 entering it. This qualitatively explains the increase in capture probability with increasing noise in the chemotaxis-dominated regime.

A rough measure of *P*_*cap *_for low noise can be obtained by the following argument. In the deterministic case, the capture radius is determined by the initial cell position (denoted *d *in the previous section), for which the distance of closest approach equals the sum of the cell and obstacle radii, i.e. *r*_*min *_= *a *+ *R*. The addition of noise to the system enables a cell to be captured for *r*_*min *_> *a *+ *R*. Consider a cell with an initial position that places it outside the deterministic capture radius. By the time a cell has arrived at the obstacle's equator (where the distance of closest approach occurs), the fluctuations in its position are roughly *δ x *= 2Dδt
 MathType@MTEF@5@5@+=feaafiart1ev1aaatCvAUfKttLearuWrP9MDH5MBPbIqV92AaeXatLxBI9gBaebbnrfifHhDYfgasaacH8akY=wiFfYdH8Gipec8Eeeu0xXdbba9frFj0=OqFfea0dXdd9vqai=hGuQ8kuc9pgc9s8qqaq=dirpe0xb9q8qiLsFr0=vr0=vr0dc8meaabaqaciaacaGaaeqabaqabeGadaaakeaadaGcaaqaaiabikdaYiabdseaeHGaciab=r7aKjabdsha0bWcbeaaaaa@31E7@ = 2DL0/αg
 MathType@MTEF@5@5@+=feaafiart1ev1aaatCvAUfKttLearuWrP9MDH5MBPbIqV92AaeXatLxBI9gBaebbnrfifHhDYfgasaacH8akY=wiFfYdH8Gipec8Eeeu0xXdbba9frFj0=OqFfea0dXdd9vqai=hGuQ8kuc9pgc9s8qqaq=dirpe0xb9q8qiLsFr0=vr0=vr0dc8meaabaqaciaacaGaaeqabaqabeGadaaakeaadaGcaaqaaiabikdaYiabdseaejabdYeamnaaBaaaleaacqaIWaamaeqaaOGaei4la8ccciGae8xSdeMaem4zaCgaleqaaaaa@34F2@, implying that *r*_*min*_~(*a *+ *R*) + 2DL0/αg
 MathType@MTEF@5@5@+=feaafiart1ev1aaatCvAUfKttLearuWrP9MDH5MBPbIqV92AaeXatLxBI9gBaebbnrfifHhDYfgasaacH8akY=wiFfYdH8Gipec8Eeeu0xXdbba9frFj0=OqFfea0dXdd9vqai=hGuQ8kuc9pgc9s8qqaq=dirpe0xb9q8qiLsFr0=vr0=vr0dc8meaabaqaciaacaGaaeqabaqabeGadaaakeaadaGcaaqaaiabikdaYiabdseaejabdYeamnaaBaaaleaacqaIWaamaeqaaOGaei4la8ccciGae8xSdeMaem4zaCgaleqaaaaa@34F2@. The distance *L*_0 _is the length of the cell's path from its initial position to the point at which it reaches the obstacle's equator; this is roughly equal to 3 in our case. Given the new *r*_*min*_, one can compute *P*_*cap *_as previously done for the deterministic case. Note that this rough calculation overestimates *P*_*cap*_; this is because we have not taken into account the fact that some cells initially within the capture radius will escape capture, as explained in the previous paragraph. The stochastic correction to *r*_*min *_is relatively more significant for small cell radii than for larger ones; this qualitatively explains why there is hardly any change in *P*_*cap *_for *a *≳ 0.4 over four orders of magnitude of noise, but a marked change for smaller values of *a*.

In our simulations we have kept the obstacle radius *R *fixed at unity. In general we find that the effect of increasing *R *(all other factors constant) is qualitatively the same as decreasing the cell radius *a*. However, note that whereas in the deterministic case the behavior was determined exclusively by the ratio *a*/*R*, this is not the case here, except in the limit of low noise.

We have also investigated the effect of noise on the motion of a cell around a perfectly absorbing obstacle. As for the non-absorbing case, we find that there are two distinct regimes: chemotaxis-dominated and diffusion-dominated. For the first regime, the capture probability increases with noise strength, whereas in the second the opposite effect occurs. The reasons are the same as for the non-absorbing case. One peculiarity of the absorbing case is the following. For the deterministic case, the capture probability is zero for cells smaller than a critical radius and greater than zero otherwise (see Fig. 5b). Low noise lowers this critical threshold. It is also generally the case that noise has less effect on the capture probabilities for the absorbing than for the non-absorbing obstacle case. This is because cells passing around absorbing obstacles tend to remain further from the obstacle than if the obstacle was non-absorbing, as is clear from the trajectories illustrated in Fig. 2 and Fig. 3.

We finish this section by noting that if we had to consider the effect of noise on the capture probability of a cell in the presence of many obstacles, then the situation is considerably more complex. In particular, the results of this section would only hold in the more general case if the concentration of obstacles was small.

### Efficiency of chemotaxis in a multi-obstacle space

Under *in vivo *conditions, chemotactic cells have to navigate to the chemotactic source by avoiding various kinds of obstacles. The question we want to address in this section is: what is the mean free path of a chemotactic cell under *in vivo *conditions? In other words, over what spatial distances is chemotaxis an efficient process for guiding cells from one location to another?

To answer such a question, the most general scenario to consider would be a random 3D distribution of obstacles. Let the obstacles be of the non-absorbing kind and let the mean obstacle separation be significantly greater than the obstacle radius. The latter assumption guarantees that the field around any given obstacle is decoupled from the effects of nearby ones. This assumption will enable us to use the results derived in previous sections. We restrict ourselves to deterministic cell movement.

The average distance traveled by a cell before permanent capture is conceptually the same as the mean free path of a gas molecule, which is usually estimated from kinetic theory [22]. Consider a very thin slab of space of cross-sectional area *L*^2 ^and infinitesimal width *dz*, in which obstacles are randomly distributed with a number density *ρ*_*o*_. The effective cross-section for capture by each obstacle, is *π*rcap2
 MathType@MTEF@5@5@+=feaafiart1ev1aaatCvAUfKttLearuWrP9MDH5MBPbIqV92AaeXatLxBI9gBaebbnrfifHhDYfgasaacH8akY=wiFfYdH8Gipec8Eeeu0xXdbba9frFj0=OqFfea0dXdd9vqai=hGuQ8kuc9pgc9s8qqaq=dirpe0xb9q8qiLsFr0=vr0=vr0dc8meaabaqaciaacaGaaeqabaqabeGadaaakeaacqWGYbGCdaqhaaWcbaGaem4yamMaemyyaeMaemiCaahabaGaeGOmaidaaaaa@333B@, where *r*_*cap *_is the capture radius as defined by Eq. (7). Then the obstacles present a total capture area equal to (*π*rcap2
 MathType@MTEF@5@5@+=feaafiart1ev1aaatCvAUfKttLearuWrP9MDH5MBPbIqV92AaeXatLxBI9gBaebbnrfifHhDYfgasaacH8akY=wiFfYdH8Gipec8Eeeu0xXdbba9frFj0=OqFfea0dXdd9vqai=hGuQ8kuc9pgc9s8qqaq=dirpe0xb9q8qiLsFr0=vr0=vr0dc8meaabaqaciaacaGaaeqabaqabeGadaaakeaacqWGYbGCdaqhaaWcbaGaem4yamMaemyyaeMaemiCaahabaGaeGOmaidaaaaa@333B@) *ρ*_*o *_*L*^2 ^*dz*; thus it follows that the probability of a cell being captured as it passes through the slab of space is equal to:

P=(πrcap2)ρoL2dzL2=(πrcap2)ρodz.     (18)
 MathType@MTEF@5@5@+=feaafiart1ev1aaatCvAUfKttLearuWrP9MDH5MBPbIqV92AaeXatLxBI9gBaebbnrfifHhDYfgasaacH8akY=wiFfYdH8Gipec8Eeeu0xXdbba9frFj0=OqFfea0dXdd9vqai=hGuQ8kuc9pgc9s8qqaq=dirpe0xb9q8qiLsFr0=vr0=vr0dc8meaabaqaciaacaGaaeqabaqabeGadaaakeaacqWGqbaucqGH9aqpdaWcaaqaamaabmaabaacciGae8hWdaNaemOCai3aa0baaSqaaiabdogaJjabdggaHjabdchaWbqaaiabikdaYaaaaOGaayjkaiaawMcaaiab=f8aYnaaBaaaleaacqWGVbWBaeqaaOGaemitaW0aaWbaaSqabeaacqaIYaGmaaGccqWGKbazcqWG6bGEaeaacqWGmbatdaahaaWcbeqaaiabikdaYaaaaaGccqGH9aqpdaqadaqaaiab=b8aWjabdkhaYnaaDaaaleaacqWGJbWycqWGHbqycqWGWbaCaeaacqaIYaGmaaaakiaawIcacaGLPaaacqWFbpGCdaWgaaWcbaGaem4Ba8gabeaakiabdsgaKjabdQha6jabc6caUiaaxMaacaWLjaWaaeWaaeaacqaIXaqmcqaI4aaoaiaawIcacaGLPaaaaaa@5A30@

Setting *P *= 1 gives us the typical distance traveled before capture, *λ*:

λ=1πρorcap2=1+δπρoR2[(1+δ)3−1],     (19)
 MathType@MTEF@5@5@+=feaafiart1ev1aaatCvAUfKttLearuWrP9MDH5MBPbIqV92AaeXatLxBI9gBaebbnrfifHhDYfgasaacH8akY=wiFfYdH8Gipec8Eeeu0xXdbba9frFj0=OqFfea0dXdd9vqai=hGuQ8kuc9pgc9s8qqaq=dirpe0xb9q8qiLsFr0=vr0=vr0dc8meaabaqaciaacaGaaeqabaqabeGadaaakeaaiiGacqWF7oaBcqGH9aqpdaWcaaqaaiabigdaXaqaaiab=b8aWjab=f8aYnaaBaaaleaacqWGVbWBaeqaaOGaemOCai3aa0baaSqaaiabdogaJjabdggaHjabdchaWbqaaiabikdaYaaaaaGccqGH9aqpdaWcaaqaaiabigdaXiabgUcaRiab=r7aKbqaaiab=b8aWjab=f8aYnaaBaaaleaacqWGVbWBaeqaaOGaemOuai1aaWbaaSqabeaacqaIYaGmaaGcdaWadaqaamaabmaabaGaeGymaeJaey4kaSIae8hTdqgacaGLOaGaayzkaaWaaWbaaSqabeaacqaIZaWmaaGccqGHsislcqaIXaqmaiaawUfacaGLDbaaaaGaeiilaWIaaCzcaiaaxMaadaqadaqaaiabigdaXiabiMda5aGaayjkaiaawMcaaaaa@5796@

where *δ *= *a*/*R*. An interesting consequence of this formula is that for small cells (*a *≪ *R*), *λ *is proportional to 1/*R*. If we did not take account of the spatial perturbations in the chemical field due to the obstacle, the capture radius *r*_*cap *_would simply be equal to *R*, implying that *λ *∝ 1/*R*^2^. It is also easy to show that since the fractional change in the number density of cells after they have passed through the slab is proportional to *P*, the spatial distribution of cells has to be exponential: *ρ*_*c *_∝ *e*^-*z*/*λ*^, where *ρ*_*c *_is the number density of cells.

Note that the above estimates are only valid for low obstacle number density. It is not possible to derive *λ *for the stochastic case since there are no explicit expressions for the capture radius. For the absorbing obstacle case, *r*_*cap *_= 0 for cells smaller than a critical size and greater than zero otherwise. This implies that *λ *= ∞ for cells below the critical size and finite otherwise.

## Conclusion

The main aim of this study was to investigate how cells avoid obstacles in *in vivo *environments: do they need a special obstacle-sensing mechanism to follow a chemotactic signal efficiently in an obstacle-ridden spatial region? In this article, we have investigated by means of a simple model, the movement of a chemotactic cell when an obstacle is placed in its direct path of motion towards a chemotactic source. The physical presence of the obstacle perturbs the chemical field near its surface. A cell on a direct collision course with the obstacle can in many cases avoid it by simply following the perturbed chemical gradient in its vicinity. The ability to do so depends strongly on two factors: the cell-to-obstacle size ratio and the chemical absorbing properties of the obstacle.

If the obstacle does not absorb any chemical, then cells of all sizes have a non-zero probability of avoiding it. The probability is very small for cells comparable in size to the obstacle and only appreciable for cells with radii smaller than approximately a quarter of the obstacle's radius.

If the obstacle sequesters chemical molecules at its surface, then the situation is very different. In this case, cells smaller than a certain critical size always avoid the obstacle. This critical size can be comparable to or even larger than the obstacle size, meaning that even large cells on a direct collision path with the obstacle can easily avoid it by simply following the perturbed gradient.

For both cases, we find that noise (as measured by the cell diffusion coefficient) decreases the chances of a cell avoiding an obstacle if the dynamics are chemotaxis-dominated and increases its chances if noise-dominated. By chemotaxis-dominated we mean that the cell's velocity is primarily determined by the chemical gradient, whereas noise-dominated means that the cell's motion appears to be similar to a random walk, though it is weakly biased in the direction of the chemical gradient. Interestingly, a cell is least successful in escaping an obstacle when chemotaxis and noise contribute approximately equally to its motion. Note that although large noise enhances the cell's obstacle avoidance ability, it simultaneously reduces its ability to follow the direction dictated by the chemical gradient. Thus, overall, cells with low noise, i.e. small diffusion coefficients, are most advantaged in avoiding obstacles and successfully following the chemical pre-pattern.

We also find that the trajectories of cells with linear chemotactic responses around non-absorbing obstacles in a static fluid are exactly of the same mathematical form as the streamlines of a non-viscous fluid past a static obstacle. This means that the two cases are physically indistinguishable. This equivalence does not hold for the case of an absorbing obstacle, or for non-linear or delayed chemotactic responses.

Throughout our study we have implicitly ignored short-range hydrodynamic interactions between the cell and the obstacle and simply modeled the interaction of the two by assuming that a cell irreversibly adheres to an obstacle upon mechanical contact. The fate of a real cell when it touches some obstacle depends on the complex interfacial forces between the two. A possible scenario is that upon encounter with an obstacle, a cell temporarily adheres, but intrinsic noise in its motion will eventually enable it to leave the obstacle's surface. However, note that low noise can only lead to temporary and frequent sticking and unsticking of the cell about the point of its first capture, so a captured cell will take a significantly long time to pass the obstacle in such circumstances. Chemical gradients are set up for some finite period of time; if the time required for a cell to pass an obstacle is comparable to or longer than this time, than the cell would effectively be counted as captured. Thus our conclusion is that for low noise, reversible adhesion (temporary capture) leads to the same results we derived in this article for irreversible adhesion (permanent capture).

In conclusion we have shown, by considering the underlying physics, that chemotactic cells, in many cases can avoid obstacles by simply following the spatially perturbed chemical gradients around them. This may explain why specialized biological mechanisms for avoiding obstacles are only known for a handful of cells and organisms.
